# Reducing inequities in maternal and child health in rural Guatemala through the CBIO+ Approach of Curamericas: 6. Management of pregnancy complications at Community Birthing Centers (*Casas Maternas Rurales*)

**DOI:** 10.1186/s12939-022-01758-6

**Published:** 2023-02-28

**Authors:** Elijah T. Olivas, Mario Valdez, Barbara Muffoletto, Jacqueline Wallace, Ira Stollak, Henry B. Perry

**Affiliations:** 1 Health Systems Program, Department of International Health, Johns Hopkins Bloomberg School of Public Health, Baltimore, Maryland USA; 2 Curamericas/Guatemala, Calhuitz, San Sebastián Coatán, Huehuetenango, Guatemala; 3 Curamericas Global, Raleigh, North Carolina USA; 4 Independent consultant, Baltimore, Maryland USA

**Keywords:** Pregnancy complications, Emergency obstetric care, Obstetrical referral compliance, Maternal health, Child health, Community health, Primary health care, Community-based primary health care, Implementation research, Census-Based, Impact-Oriented Approach, Care Groups, Community birthing centers, Guatemala, Equity, Curamericas Global, Curamericas/Guatemala, Maya culture

## Abstract

**Background:**

In Guatemala, Indigenous women have a maternal mortality ratio over twice that of non-Indigenous women. Long-standing marginalization of Indigenous groups and three decades of civil war have resulted in persistent linguistic, economic, cultural, and physical barriers to maternity care. Curamericas/Guatemala facilitated the development of three community-built, -owned, and -operated birthing centers, *Casas Maternas Rurales* (referred to here as Community Birthing Centers), where auxiliary nurses provided physically accessible and culturally acceptable clinical care. The objective of this paper is to assess the management of complications and the decision-making pathways of Birthing Center staff for complication management and referral. This is the sixth paper in the series of 10 articles. Birthing centers are part of the Expanded Census-based, Impact-oriented Approach, referred to as CBIO+.

**Methods:**

We undertook an explanatory, mixed-methods study on the handling of pregnancy complications at the Birthing Centers, including a chart review of pregnancy complications encountered among 1,378 women coming to a Birthing Center between 2009 and 2016 and inductively coded interviews with Birthing Center staff.

**Results:**

During the study period, 1378 women presented to a Birthing Center for delivery-related care. Of the 211 peripartum complications encountered, 42.2% were successfully resolved at a Birthing Center and 57.8% were referred to higher-level care. Only one maternal death occurred, yielding a maternal mortality ratio of 72.6 maternal deaths per 100,000 live births. The qualitative study found that staff attribute their successful management of complications to frequent, high-quality trainings, task-shifting, a network of consultative support, and a collaborative atmosphere.

**Conclusion:**

The Birthing Centers were able to resolve almost one-half of the peripartum complications and to promptly refer almost all of the others to a higher level of care, resulting in a maternal mortality ratio less than half that for all Indigenous Guatemalan women. This is the first study we are aware of that analyzes the management of obstetrical complications in such a setting. Barriers to providing high-quality maternity care, including obtaining care for complications, need to be addressed to ensure that all pregnant women in such settings have access to a level of care that is their fundamental human right.

## Background

In isolated rural areas of low-resourced countries where health care facilities are far away, viable alternatives to home births are needed. In the northwest corner of Guatemala where the non-governmental organization Curamericas/Guatemala has been working for two decades to strengthen health services for mothers and children, Community Birthing Centers staffed by lower-level health workers have been established to provide a safe yet still culturally appropriate alternative [1]. Here we assess the frequency and management of peri-partum complications occurring in these Birthing Centers. We are not aware of any similar studies to date carried out in Community Birthing Centers where higher-level referral care is difficult to access.

In rural areas of low﻿- and middle-income countries (LMICs), home births remain commonplace: There are 30 LMICs in which more than half of births in rural areas take place in the home [2]. Research has linked Guatemala’s relatively high Indigenous maternal mortality ratio (MMR) to home births [3]. Indigenous women in Guatemala suffer an MMR more than twice that of their non-Indigenous counterparts (166 vs. 78) [4]. Elsewhere in this series, we report that Indigenous Maya women who give birth at home in the isolated highlands of the Department of Huehuetenango have an eight-fold greater risk of maternal mortality than women who give birth in a facility [5]. The 2011 National Study of Maternal Mortality further attributed this disparity to the historic economic and cultural marginalization of and discrimination against the country’s Indigenous population, and the associated failure of the formal health system to adequately serve it [4]. Government clinics and hospitals are often too distant and costly for isolated, poor, mountain-dwelling populations to access. As these populations are composed almost exclusively of Indigenous people of Maya descent, the lack of cultural accessibility is an equally strong barrier, with Maya families often shunning available government facilities because the staff tend not to speak their language, because treatment is often disrespectful, and because their cultural birthing practices are scorned and even prohibited [6–8]. Many see these problems as violations of human rights as well as violations of Guatemalan law and its constitution [4, 9]. The Ministry of Health and Social Assistance (*Ministerio de Salud Pública y Asistencia Social*, MSPAS) has attempted to address these problems by training and supporting traditional birth attendants (*comadronas*) and by improving the cultural acceptability of maternal health care services provided in public health facilities, but these initiatives have been met with limited success [9].

This paper is the sixth in our series of 10 papers describing the effectiveness of the Expanded Census-based, Impact-oriented Approach (CBIO+) in improving the health and well-being of mothers and children in rural Guatemala. CBIO+ builds on the traditional CBIO approach by incorporating Care Croups and Community Birthing Centers, referred to in the programs of Curamericas/Guatemala as *Casas Maternas Rurales.*

The first paper introduces CBIO+ and the Curamericas/Guatemala Maternal and Child Health Project, 2011 to 2015 (hereafter referred to as the Project) [10], and the second paper describes the implementation research methodology [11]. The third paper describes increases in population coverage of key interventions [12]. The fourth paper describes changes in nutritional status [13] and the fifth paper provides an assessment of levels, causes, and changes in mortality [5]. Subsequent papers in the series address women’s empowerment [14, 15], stakeholder perspectives concerning the effectiveness of CBIO+ [16], and cost-effectiveness and policy implications [17].

One of the important features of CBIO+ is the registration of vital events through frequent contacts with all homes [18] and another is the development of Community Birthing Centers [19]. In the previous paper in this series, we documented that the Project Area has one of the highest documented levels of maternal mortality in the Western hemisphere of which we are aware: during the period of the Project’s operation, the MMR was 477 per 100,000 live births [5], more than five times higher than the national levels reported by UNICEF [20] and Every Mother Counts [21] of 95 deaths per 100,000 live births and 73 deaths per 100,000 live births, respectively, and almost three times the MMR for Indigenous women in Guatemala nationally [4]. At the outset of the Project, 82%﻿ of births took place in the home [12], as did 94% of maternal deaths [5]. Even when obstetrical complications are promptly recognized in the Project Area, arrival at a hospital where these can be managed requires 4 hours of travel. Home deliveries were associated with an eight-fold increased risk in mortality [5]. Thus, in this setting location of delivery and time to obtaining emergency care are critical factors in ensuring maternal survival when complications arise.

One of the Project’s strategies for improving the health and well-being of mothers and children in the Project Area was to provide a feasible and safer alternative to home births by developing Community Birthing Centers. As described further in Paper 1 [10] and elsewhere [19], these are 2–3 room buildings constructed by the community and staffed 24/7 with auxiliary nurses and support women where women can come with their *comadrona* to obtain a clean and safe delivery and where, if a complication is detected, an emergency transport system can be activated quickly for a hospital referral.

This paper addresses the management of obstetrical complications that arose among women who came to a Birthing Center to give birth. We employed mixed methods to: (1) quantitatively investigate the complications encountered and managed at the Birthing Centers, (2) examine reasons for the Birthing Centers’ successful management of pregnancy complications, and (3) explore the challenges encountered by staff in the management of obstetrical complications. Table 1 presents our research questions. The findings of the quantitative study informed the follow-up qualitative investigation into the complex processes and situations that surround the management and referral of complications.

**Table 1:** Research questions

Data type	Research question
Quantitative	1. How many complications are attended at the Birthing Centers? Is the number growing? 2. What are the complications? 3. What percentage of deliveries attended at the Birthing Centers involve complications? 4. Which types of complications are resolved at the Birthing Centers? Which types of complications are referred? 5. What are the outcomes for the women with complications, both resolved and referred?
Qualitative	1. Why have the Birthing Centers been successful at managing complications? 2. Why are cases that should have been referred to a higher-level facility instead resolved by staff in the Birthing Center? 3. Why do women from outside of Birthing Center partner communities tend to present with more severe complications than women from partner communities?

## Methods

### Setting

As described in Paper 1 in this series [10], this study took place in the remote, mountainous, western highlands of the Department of Huehuetenango. The Department’s population is more than two-thirds Indigenous Maya. In 2011, 60.5% of the department’s population lived in poverty and 9.6% lived in extreme poverty [22]. In 2013, the departmental MMR, as reported by official government statistics, was 277 per 100,000 live births [23]. The MSPAS estimated that 92% of maternal deaths would be preventable with adequate antenatal, intrapartum, and post-partum care. The leading cause of maternal death in the department in 2013 was hemorrhage (49%), followed by pre-eclampsia/eclampsia (32%) and sepsis (13%). In the municipalities of San Sebastián Coatán and San Miguel Acatán﻿, the locations of this study, the official 2013 MMRs were 552 and 379, respectively – among the highest reported in Latin America [23]. Figure 1 in Paper 2 in this series [11] shows the location of the three Birthing Centers where the complications described in this paper were managed.

To address these disparities, in 2002 Curamericas/Guatemala, a non-governmental organization (NGO) registered in Guatemala, partnered with the US-based NGO Curamericas Global to reduce maternal and child mortality in three municipalities in the Huehuetenango Department. Curamericas/Guatemala’s main strategies are (1) community mobilization and empowerment; (2) health outreach to promote improved household health behaviors and appropriate utilization of health services; and (3) *Casas Maternas Rurales* (hereafter referred to as Community Birthing Centers or as *Casas* by respondents) to achieve this goal. Between 2009 and 2014, Curamericas/Guatemala partnered with 26 local Chuj and Akateko Maya communities to establish three Birthing Centers serving a population of 8,702 in the rural municipalities of San Sebastián Coatán and San Miguel Acatán in the Huehuetenango Department.

Unlike maternity waiting homes, where near-term women with high-risk pregnancies travel before the onset of labor to a far-away hospital and reside in a waiting home adjacent to the hospital until they deliver [24], Birthing Centers serve a local population and are community-built, -owned, and -operated. They provide high-quality maternal and neonatal health services, including deliveries [1]. The Birthing Centers developed by Curamericas/Guatemala serve are located in remote communities, distant from higher-level health facilities. They provide clean, safe spaces for women to deliver according to their cultural preferences.

Each of the three Birthing Centers was staffed by three auxiliary nurses and two support women (*Mujeres de Apoyo*), who were trained and supported by an obstetric nurse supervisor who was based in Calhuitz. Each woman giving birth was welcome to have her own *comadrona* join her and participate in the birthing process. *Comadronas* have become enthusiastic supporters of the Birthing Centers and have encouraged their clients to deliver there [10].

The auxiliary nurses received training from the Project Director (MV), MSPAS physicians, the obstetric nurse supervisor, and visiting health professionals from the United States (including faculty from the Duke University School of Nursing). They were also able to attend special one-week training courses on management of obstetrical emergencies and Kangaroo Mother Care given by Project Concern International in the city of Huehuetenango. The training included the components of a safe delivery; Essential Newborn Care, which included clean cord care, immediate thermal care, and immediate/exclusive breastfeeding; the use of partographs; the Active Management of the Third Stage of Labor, including the administration of oxytocin after the delivery of the baby; as well as neonatal resuscitation (using Ambu bag and mask) and stabilization/resolution of any neonatal complications.

The Birthing Centers were financially accessible: patients pay a base fee of approximately US$20 that covers all medical supplies, staff and use of the facility. There is an additional fee of approximately US$7 for food and cleaning of linens. Alternatively, the families could provide these themselves. Pregnant women and their families also have the option of making a one-time payment of approximately US$13 as insurance to assist with the cost of emergency transport to the hospital if needed. If the insurance fee was paid, the insurance covered one-half of the US$150 cost of transport to the MSPAS referral hospital in the city of Huehuetenango. While the approximately US$75 net cost to the insured family for transport was high in this context of poverty, most families of women in need of emergency transport managed to gather this sum.

In 2012, when there was only one operating Birthing Center, only 30% of births in the 26 partner communities occurred at a Birthing Center or higher-level health facility. Our results show that between January 2014 and June 2016, with the addition of two new Birthing Centers, facility deliveries increased to 80% of all births in the 26 partner communities and the percentage of births occurring in the Birthing Centers increased to 70% (Table 2) [25]. From the first year that all three Birthing Centers were operational (2014) through June 2016, Project vital events data showed no maternal deaths in the partner communities among the 831 live births that occurred there [25]. The apparent elimination of maternal deaths in partner communities in association with the Birthing Center*s*’ steadily increasing utilization strongly suggests that the Birthing Centers were directly contributing to the reduction in maternal mortality. Since obstetric complications are the most common proximate cause of maternal mortality, we implemented this study to investigate the handling of complications by the Birthing Center staff. As far as we know, this is the first published study that focuses on the management of obstetrical complications in a community birthing center staffed by lower-level health workers who are not professional midwives.

**Table 2:** Number of deliveries by place of delivery and year among 26 partner communities

Location of delivery	2014	2015	2016 (January 1 through June 30)	Total
Birthing Center deliveries	166 (53.4%)	210 (60.2%)	120 (70.2%)	496
Non-Birthing Center health facility deliveries^a^	45 (14.5%)	29 (8.3%)	16 (9.4%)	90
Home deliveries	100 (32.2%)	110 (31.5%)	35 (20.5%)	245
**Total**	**311 (100.0%)**	**349 (100.0%)**	**171 (100.0%)**	**8 31**

^a^These took place at a government health facility in the Project Area or at a hospital or private clinic outside of the Project Area. There were not any private health facilities in the Project Area

### Data collection process

We utilized a two-pronged, explanatory mixed-methods approach to data collection. Data from paper registers maintained by Birthing Center staff documenting complications were first transferred to Microsoft Excel, recording every complication captured in the clinical delivery records of the three Birthing Centers. Complications tracked include those immediately before, during, and immediately after birth (peripartum complications). The registers include demographic information on clients, their condition and the care received, and, in the case of referrals, the referral facility, services provided, and outcome. A descriptive analysis of data was used to answer the quantitative research questions shown in Table 1, utilizing Microsoft Excel. Our analysis incorporated registry data from the initiation of each of the three Birthing Centers, beginning in May 2009, through June 2016, as shown in Table 3.

**Table 3:**
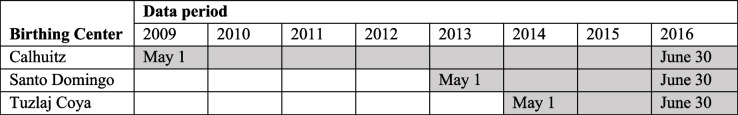
Registry data collection period

After the descriptive analysis of the registry data was completed, a qualitative investigation was designed and conducted to answer the research questions provoked by the quantitative findings. The study utilized a self-administered written questionnaire followed by a focus group discussion (FGD) with staff from all three operating Birthing Centers along with a separate key informant interview with the Project Director (Table 4).

**Table 4:** Qualitative data collection methods

Method	Topics investigated	Participants	Date and location
Self-administered questionnaire	1. Staff training and evaluation at the Birthing Centers 2. ﻿Staff roles during births at the Birthing Centers 3. Decision to resolve or refer complications 4. Perceived reasons for a family to refuse a referral to a higher-level health facility 5. Differences between partner and non-partner communities 6. Vision as to how Birthing Centers will change going forward, and recommendations	**Supervisory nurses:** All Birthing Centers (3) **Auxiliary nurses:** Birthing Center in Santo Domingo (1) Birthing Center in Calhuitz (1) Birthing Center in Tuzlaj (1) **Support women:** Birthing Center in Santo Domingo (2) Birthing Center in Tuzlaj (2) Birthing Center in Calhuitz (2)	Submitted electronically between November 13 & 17, 2016﻿ Completed at﻿ Birthing Centers in Calhuitz, Tuzlaj, and Santo Domingo
Focus group discussion	1. Decision to resolve or refer complications 2. The effect of refused referrals and how they can be prevented or avoided 3. Differences in patterns of complications between women who come to the Birthing Centers from partner communities versus those from non-partner communities 4. Relationship between Birthing Center*s* and the MSPAS 5. Vision as to how Birthing Centers will change going forward, and recommendations	**Supervisory nurses:** All Birthing Centers (3) **Auxiliary nurse:** Birthing Center in Santo Domingo (1) **Support women:** Birthing Center in Tuzlaj (1) and Calhuitz (1)	December 12, 2016. Birthing Center headquarters in Calhuitz with Curamericas Global staff (BM) facilitating via Skype video from Raleigh, NC, USA
Key informant interview	1. Decision to resolve or refer complications 2. The effect of refused referrals and how they can be prevented or avoided 3. Differences in patterns of complications between women who come to the Birthing Centers from partner communities versus those from non-partner communities 4. Relationship between Birthing Centers and the MSPAS 5. Vision as to how Birthing Centers will change going forward, and recommendations	**Project Director**	December 20, 2016. Birthing Center headquarters in Calhuitz with Curamericas Global staff (BM) facilitating via Skype video from Raleigh, NC, USA

Information about the procedures for each collection method is described below.

### Self-administered questionnaires

In November 2016, the Project Director coordinated with staff to complete the questionnaires using Microsoft Word on the Project’s computer. Questionnaires were returned to the investigators via email. The completed questionnaires were not anonymous but were confidential, with only the investigators having access to them. The questionnaire was filled out by 12 staff who worked at the three Birthing Centers*.* The questionnaire asked staff members to provide information about the training they received, workplace procedures, and the factors they weighed when determining whether to refer or resolve an obstetric complication (e.g., the type of complication and the severity of the complication). The questionnaire began with a description of its purpose. Staff were not required to answer all questions. Staff were instructed to send their responses directly to Curamericas Global. Responses were saved on a password-protected computer and made available only to the Curamericas Global staff members who were working on the study.

### Focus group and key informant interview

The FGD was held the next month via Skype. At least one staff member was present from each level of staff in each Birthing Center. The Project Director was intentionally left out of the FGD to encourage Birthing Center staff to speak openly about their experiences. A subsequent key informant interview was conducted with the Project Director. The FGD and key informant interview were conducted in Spanish, recorded, and then later transcribed and translated to English.

The focus group and key informant interviews began with the reading of a consent statement in Spanish. The statement provided an overview of the study purpose and how responses would be used. The participants were informed that the discussion would be recorded, transcribed in Spanish, and saved in a secure location and that the information they provided would be kept confidential and accessible only to Curamericas Global staff on a password-protected computer. Consent from participants was documented in the recording. Curamericas Global adhered to the statements in the consent form and further removed identifying information in the paper by removing not only staff names, but also staff positions and locations from illustrative quotes.

During the FGD, Birthing Center s﻿taff were asked about their training and about decision-making pathways for determining if an obstetric complication should be managed onsite, as well as their course of action if a referral was rejected. Staff provided their perceptions of the underlying rationale for the decisions and actions taken by mothers and their families. To maintain anonymity, the quotations cited in the qualitative findings that were given by the Project Director are labeled as a Birthing Center staff member along with the quotations arising from the FGDs.

### Data analysis

After removing all respondent names, the questionnaire responses and transcriptions of the FGD were analyzed by two investigators (BM and IS) using Microsoft Excel and Microsoft Word. Responses were coded inductively using systematic, thematic coding. All responses were matched to a predetermined codebook, with new codes created for additional themes as they emerged. Each participant’s entire response was entered into an Excel matrix and disaggregated by research question. Predetermined response themes relating directly to the questions were identified and then analyzed. Responses were further organized by the additional themes discovered during analysis. Responses to the questionnaires, FGDs, ﻿and interviews were analyzed individually and then combined for this report, with many responses supporting insights found in the other methods. Data were triangulated by combining a summary of the responses to each research question by qualitative method, so that all the responses under each method were combined. Triangulation of data helped to create more comprehensive answers to research questions and provide further insight into the themes that emerged.

## Results

### Quantitative findings

In this section, we describe the types of maternal complications encountered by Birthing Center staff during labor, delivery, and the immediate post-partum period. Between May 1, 2009 and June 30, 2016, 1,378 women presented to the Birthing Centers for intrapartum care. During the study period, 211 women experienced at least one peripartum complication – a peripartum complication rate of 15.3% (Table 5). Birthing Center staff were able to successfully resolve 89 (42.2%) of these peripartum complications, while referring 122 (57.8%) to higher-level care.

**Table 5:** Birthing Center peripartum complications

Indicator	2009 (starting May 1)	2010	2011	2012	2013	2014	2015	2016 (Jan 1 through June 30)	Total
Peripartum patients	64	91	94	108	206	264	340	211	**1,378**
Peripartum complications	13	13	29	15	24	34	53	30	**211**
Peripartum complication rate	20.3%	14.3%	30.9%	13.9%	11.7%	12.9%	15.6%	14.2%	**15.3%**

The types of complications encountered were examined in detail (Table 6). The most common complications diagnosed include footling breech presentation (25.1% of all complications), labor dystocia (22.3%), hemorrhage (16.6%), and pre-eclampsia/eclampsia (11.4%). For each classification of complication, the percentages that were successfully resolved and referred were calculated, as shown in Table 6. The Birthing Center staff’s successful resolution of a complication varied widely depending on the type of complication. For example, only one case (4.2%) of pre-eclampsia/eclampsia was resolved at the Birthing Centers, with all other cases (95.8%) being referred, while 90.6% of footling breech presentations were successfully delivered at the Birthing Centers.

**Table 6:** Pregnancy complications diagnosed at the Birthing Centers

Complication	Total		Referred (%)	Resolved (%)	% of complications (*n* = 211)
Malpresentation (footling breech)	53		5 (9.4%)	48 (90.6%)	25.1%
Labor dystocia/obstructed labor	47		36 (76.6%)	11 (23.4%)	22.3%
Hemorrhage	35		24 (68.6%)	11 (31.4%)	16.6%
Pre-eclampsia/eclampsia	24		23 (95.8%)	1 (4.2%)	11.4%
Malpresentation (transverse lie)	21		20 (95.2%)	1 (4.8%)	10.0%
Retained placenta	13		2 (15.4%)	11 (84.6%)	6.2%
Pre-term labor	8		5 (62.5%)	3 (37.5%)	3.8%
Placenta previa	2		1 (50.0%)	1 (50.0%)	0.9%
Cord prolapse	2		0 (0.0%)	2 (100.0%)	0.9%
Other	6		6 (100.0%)	0 (0.0%)	2.8%
**Total**	**211**		**122 (57.8%)**	**89 (42.2%)**	**100.0%**

Women whose complications were resolved at a Birthing Center had a survival rate of 100.0% (*n* = 89). Of the 122 women referred to a higher-level facility, one woman from a non-partner community died from complications of eclampsia. The overall survival rate for all peripartum complications was 99.5% (210 of 211). The MMR for the 1,378 women presenting at a Birthing Center for delivery was 72.6 maternal deaths per 100,000 live births.

### Qualitative findings

#### Training received by the Birthing Center staff

Birthing Center staff at all levels noted that trainings were helpful for maintaining and learning new skills, particularly for addressing complications. According to the Birthing Center staff, the comprehensive training program provided by the supervisory nurses included both initial and ongoing trainings that utilized a mix of participatory workshops, lectures, teaching videos and presentations, experience sharing, and demonstrations. One support woman (*mujer de apoyo)* estimated that around 50% of the trainings involved role-plays and simulations, with occasional demonstrations on consenting patients. All levels of Birthing Center staff reported being evaluated after trainings. They also reported that their skills and adherence to protocols were monitored quarterly using quality verification checklists.

*In the end, the Casas are a school where every day we learn something new.*

– Birthing Center staff member

#### Referring or resolving complications

Birthing Center staff indicated that they had been trained to perform a thorough assessment of all presenting complications to determine if a referral to a higher-level facility is required or if the complication could be resolved at the Birthing Center. The staff reported that the following 10 factors were important in determining whether a patient should be referred: complication type, complication severity, experience with handling the complication, training received, guidelines from the MSPAS protocol for maternity care [26], availability of a nurse supervisor, equipment available at the Birthing Center, availability of transportation, family preference, and staff members’ intuition.

The Birthing Center staff expressed that all members of the Birthing Center team, including the supervisory nurse, auxiliary nurse, and support women, took part in deliberating as to whether to refer a complication to the hospital or try to resolve it at the Birthing Center. The Birthing Center staff found that teamwork in the decision-making process and in providing treatment gave them confidence and allowed them to more effectively attend to patients and provide high-quality care.

*Our actions are better when we all take responsibility for the patients and when we consider all the factors that could help save lives; we all have different abilities.*

– Birthing Center staff member

The staff shared that the Birthing Centers utilized task shifting to identify and manage complications. Ideally, the supervisory nurse is involved whenever a complication arises; however, if she was not available, the auxiliary nurse took the lead in resolving or referring complications. Staff state that they were encouraged to seek advice from higher-level staff members during labor and delivery as needed, and that they often reached out by cell phone to their peers and MSPAS district staff to receive either advice or direct, hands-on support.

*The staff member attending to the patient does not feel alone; rather, on the contrary, they feel the support of the rest of the team.*

– Birthing Center staff member

Staff members expressed that those presenting with severe complications were almost always referred to the government hospital in the city of Huehuetenango. Staff reported choosing to resolve complications on-site at the Birthing Center only if they could clearly do so without risking the life of the mother or baby. However, knowing that the hospital was a four-to-five-hour drive over dangerous, rough mountain roads, the risk of maternal death en route to the hospital was carefully weighed against the probability of a successful resolution at the Birthing Center.

*Life will always prevail when the knowledge, courage, and satisfaction of saving lives is met with the humility to recognize our own abilities and limits.*

– Birthing Center staff member

Birthing Center staff found that when time was a critical factor, they felt compelled to make referrals to closer MSPAS clinics, which were located only 1 or 2 h away. Still, these facilities did not always have sufficient resources to resolve the complication.

*I think that, unfortunately, there have been many times that the staff have not been able to resolve the complication, like in the* [MSPAS clinic] *in Nentón* [1–2 hours away]*, and the families find that they went there in vain because they ultimately had to go to the hospital. It was a waste of time. Unfortunately, the health system in our country is very poorly structured.*

– Birthing Center staff member

#### The family’s decision to reject or accept a referral

Staff stated that they proactively prepared pregnant women to accept a referral by addressing their fears and other barriers during prenatal classes and Care Group sessions, with the hope that the family would be prepared to act in case a complication arose. Staff mentioned helping the women prepare birth plans, urging them to save money for emergency transport, and informing them of opportunities to purchase transport insurance through the Birthing Center*.* Staff also said that they directly addressed the women’s negative perceptions of hospital care and fear of mistreatment.

*We need to have the power of persuasion; we have to tell them in the prenatal classes, “Look, do not be surprised that you could possibly be treated badly. Remember that there are many patients and they are understaffed. We have to have patience. In the hospital there are sophisticated teams that will help save your life. So, what you have to have is great patience.”*

– Birthing Center staff member﻿

When a referral to a higher-level health facility was needed, staff reported that they worked with the *comadrona* and support woman to explain the need for a referral to the patient and her family in layman’s terms using the family’s native language*.* If a patient’s family rejected a referral, Birthing Center staff called upon community leaders and MSPAS officials to talk directly with the family if time permitted. Still, staff report that many families hesitated to accept a referral and estimated that, during the study period, 15% of referral recommendations were initially rejected by families.

If a family declined the recommendation, the staff faced the difficult decision regarding whether to try to resolve the complication or to refuse to provide further care. In both cases, to legally protect the Birthing Centers and their staff, staff members carefully documented instances when a family rejected a referral and when delays in the family’s decision-making increased a patient’s risk of adverse outcomes. Staff members wrote an *Acta* (a brief narrative) about the patient’s clinical condition which indicated that the family rejected and/or delayed compliance with the referral. The family then signed the *Acta* to accept responsibility for the welfare of the patient. Staff reported that it was rare for families to reject referrals after the Birthing Center had refused to provide further care.

#### Why referrals were rejected

According to staff, many families could not be persuaded to comply with a referral, forcing the staff to decide to provide care or send the woman home. Staff shared several factors underlying families’ rejection of referrals: financial constraints, fear of disrespectful treatment at the higher-level facility, fear of leaving a familiar locale, cultural traditions, the mothers’ lack of decision-making autonomy, and the devaluation of the mothers’ lives.

*The families have few resources and do not always have the funds necessary to cover transportation to the hospital. Also, the woman is not able to make the decision … Even if the patient wants to go to the hospital, the family decides if she goes or not … The patients have also expressed that the treatment they receive at [the] Casa is very good, unlike the treatment they receive at the hospital, which they feel is very bad. Another factor is the language. At the Casa there are support women who speak the same [Maya] language as the women, where in the hospital they speak only Spanish. Lastly, culture is a barrier in that here* [in the Birthing Center]*, the birth is attended to in accordance with the culture; this is not the case in the hospital.*

– Birthing Center staff member

Staff found that transportation costs can be particularly prohibitive. Few families possess or have access to vehicles and must hire local drivers for transport. Additionally, staff reported that while medical services at the government hospital in Huehuetenango are provided for free, costs for any drugs or medical supplies used are often charged to families. Staff recognized that these medical costs, combined with the cost of transport, could be catastrophic for families. Private clinics, while closer, charge (what for these families is) an exorbitant amount for services and so were, typically, financially inaccessible for families.

*In order to pay the cost of this service you have to borrow money, take out a bank loan, or even worse, sell your land or your home.*

– Birthing Center staff member

Staff members stated that families have a strong preference to give birth within the community and this preference is compounded by the poor treatment Maya people receive at hospitals. Birthing Center staff reported that hospital staff were perceived as condescending and neglectful and rarely spoke the Maya languages. Maya mothers at the hospital were often prevented from giving birth according to their customs such as utilizing the traditional birth position, attire, and prayers, and being accompanied by extended family and the *comadrona*.

*The treatment* [in the hospital] *is dehumanizing.*

– Birthing Center staff member

Furthermore, local Maya culture is patrilocal. Married women leave their homes to live with their new husbands’ families, wherein they usually have low social rank and very little decision-making autonomy. Birthing Center staff reported that the decision to accept or reject a referral was usually made not by the woman, but by her husband or in-laws, who control family finances. As the woman is an outsider within the household, her life is often not sufficiently valued by her husband’s family to warrant paying to take her to the hospital to obtain the needed care, let alone borrowing money or selling land or other precious possessions.

*At times they do not use the resources they have. They do not value her.*

– Birthing Center staff member

*Unfortunately, here in the rural areas, when a woman gets married, she becomes his family’s property. Often the father-in-law, the mother-in-law, or even the husband’s uncles decide if they are going to take her to the hospital or not. The one whose opinion matters least is the woman. The cultural barrier is very strong.*

– Birthing Center staff member

#### Delays in care seeking

Birthing Center staff members stressed that the barriers discussed above could delay decision-making for up to several hours, such that some complications had to be treated at the Birthing Center. Families used this time to deliberate with Birthing Center staff, gather or petition funds for transportation, and meet with extended family to discuss payment.

*Yes, it is frustrating … that while the families remain undecided, we are losing more and more time, when with that time they should take advantage and put us in charge of the woman.*

– Birthing Center staff member

Furthermore, staff members reported that sometimes families would intentionally delay bringing a mother to the Birthing Center until her labor had progressed or her complication had reached a critical state. Staff found that the families preferred the Birthing Centers and knew that arriving once the labor had progressed increased the likelihood that any complications that occurred would be treated on-site. Others (particularly those from non-partner communities) mistook the Birthing Center for a clinic that could treat all complications.

*Indirectly, the families use their own strategies. Many times, they already know that if they arrive on time, we are going to send the woman to the hospital, but if they arrive in a state of emergency we will have to attend to her here. This is the attitude of the people and it is also because they know the barriers they have as well as the barriers of the health system.*

– Birthing Center staff member

*By not knowing how we work, they come here seeking services thinking that we can resolve the complication … The women arrive when they already have a complication.*

– Birthing Center staff member

*These same communities lack the knowledge and think that at the Casa we are able to resolve these types of problems, and by not knowing how we work, they come here seeking services thinking that we can resolve the complication… the women arrive when they already have a complication.*

– Birthing Center staff member

*They decide to wait in their home until the woman is fully dilated and then when they arrive our only choice is to attend to the complication if one develops.*

– Birthing Center staff member

Staff members reported that, when a mother arrives too late to make a referral if a complication develops, their options are limited and they are forced to do what they can to care for the mother and her baby.

#### Emotional impact on staff

Staff members reported immense pressure from families to resolve complications in the Birthing Center, particularly when families refused referrals and there was a high risk of death if the complication was left untreated. The emotional impact on Birthing Center staff was complex. The dominant emotions that appear in their responses are fear and frustration.

*We explain a lot to the family and the majority of the time they understand the situation but still refuse to go get the health services, and we as staff feel frustrated because we can’t do anything. It also causes fear, knowing that the woman’s life is in our hands. We do everything we can do, and we do everything within our reach.*

– Birthing Center staff member

Staff members also believed that the MSPAS distrusts the Birthing Centers and feared that any mistakes, or lives lost, could result in severe repercussions.

*The biggest fear here is, for example, that* [even though] *the work we do in the Casa is good, in the end the Ministry does not really accept the Casa. They do not value the work that we do here. Now if something bad were to happen, they would quickly come out against us. This is the fear.*

– Birthing Center staff member

On the other hand, staff members also generally empathize with families’ difficult circumstances and their reluctance to accept referrals.

*Like I said, if you were to look at the cases that we treat in the Casa, the families give us reasons [for rejecting the referral] and if I were in their position, I would do the same.*

– Birthing Center staff member

## Discussion

### Synthesis of quantitative and qualitative findings

Using the information revealed in the quantitative study, the qualitative study aimed to better understand the management of complications at the Birthing Centers from the perspective of the Birthing Center staff. The results from the qualitative study centered around three main themes:

1. The processes underlying staff choosing to resolve or refer a complication
2. Reasons for families rejecting or accepting referrals
3. The emotional impact of managing complications on Birthing Center staff

The study results elucidate a decision-making process used and strategies employed by staff, as well as factors influencing whether a complication was referred or resolved. Figure 1 illustrates how these factors supported referring and resolving a complication at the Birthing Centers.

**﻿Fig. 1:**
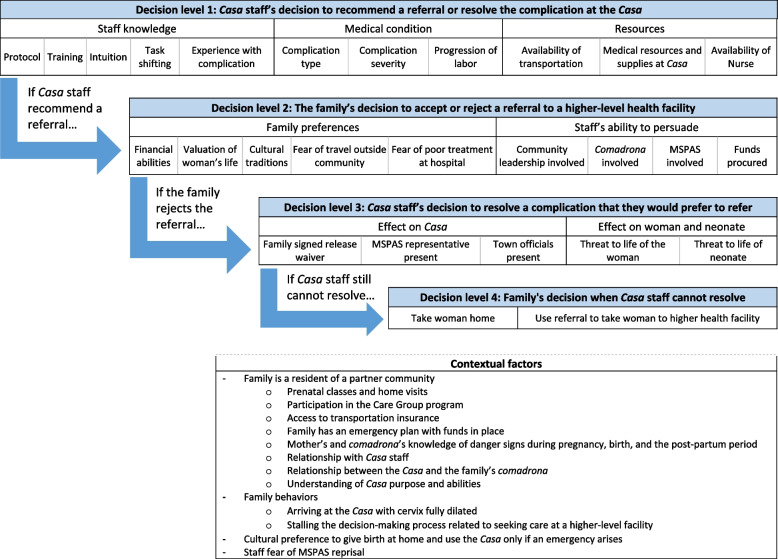
Factors influencing resolutions and referrals of birth complications. Note: The term "Casa" refers to the Birthing Center

The referral process has four main decision levels:

- **Decision level 1**: The Birthing Center staff’s decision to recommend a referral or resolve the complication at the Birthing Center.
- **Decision level 2**: The family’s decision to accept or reject the referral to a higher-level health facility.
- **Decision level 3**: If the family rejects the referral, the Birthing Center staff’s decision to resolve a case that they would prefer to refer.
- **Decision level 4**: The family’s decision if the Birthing Center staff are unable to resolve the complication.

#### Decision level 1: whether the staff recommends referral

The first level involves the Birthing Center staff’s initial decision between recommending a referral or resolving the complication on-site. Aspects of staff knowledge, the complication itself, and available resources are considered. From the interviews and questionnaires, it is evident that staff receive comprehensive training upon hire and then consistent, ongoing supportive supervision and in-service training. The training and task-shifting are supported by a strong organizational culture characterized by learning, constant improvement, and teamwork, all of which provide an environment conducive for treating and resolving complications on-site. The Birthing Centers also utilize task shifting, which empowers lower-level staff, specifically the auxiliary nurses, to play a meaningful role on the Birthing Center team and in resolving complications. This finding is supported by existing research that demonstrates similar successes in utilizing task shifting and teamwork to increase coverage, while maintaining quality, of complications management [27, 28]. Staff have set protocols on when to refer or resolve a case, but their recommendations are influenced by when the woman arrives in the progression of labor as well as the time and risk required to transport the woman to a distant, higher-level facility.

#### Decision level 2: whether the family accepts the referral

In the case that the Birthing Center staff decides to refer the patient, the family then must decide whether to accept the referral. This is influenced by a variety of family preferences, including valuation of the patient’s life and cultural traditions as well as the persuasive ability of the staff, the *comadrona*, and MSPAS representatives. This study provides greater detail into the degrees and types of barriers staff find that families face when accepting or rejecting a referral. Most of the families live in rural areas plagued by widespread poverty and limited access to health facilities. The cultural traditions of preference for home birth and for women joining their husbands’ families after marriage, their associated low status within that family, and lack of decision-making autonomy, when combined with the long history of discrimination toward the Maya people (e.g., disrespect for their culture and lack of translation services at the hospital), create a formidable constellation of barriers to families in accepting and complying with referrals. These findings are corroborated by recent studies completed in Guatemala that cite language, culture, cost, and distance as primary deterrents for Maya families in seeking health services [29–33].

As seen in Fig. 2 below, staff utilize concentric circles of support for convincing families to accept referrals. If the supervisory nurse and auxiliary nurse cannot convince a family to accept a referral, they can gain assistance from the woman’s *comadrona* and the Birthing Center support woman. If a family still resists, the Birthing Center staff can elicit support from community leaders (including the village mayor), the Village Health Committee, and local MSPAS staff, including in San Miguel Acatán the MSPAS district director, who is a Maya physician and has both Maya language mastery and the gravitas of a high-level position. Having this support from outside of the Birthing Center adds authority to the staff’s recommendations for referral and appears to be a successful mechanism for encouraging the acceptance of referrals.

**﻿Fig. 2:**
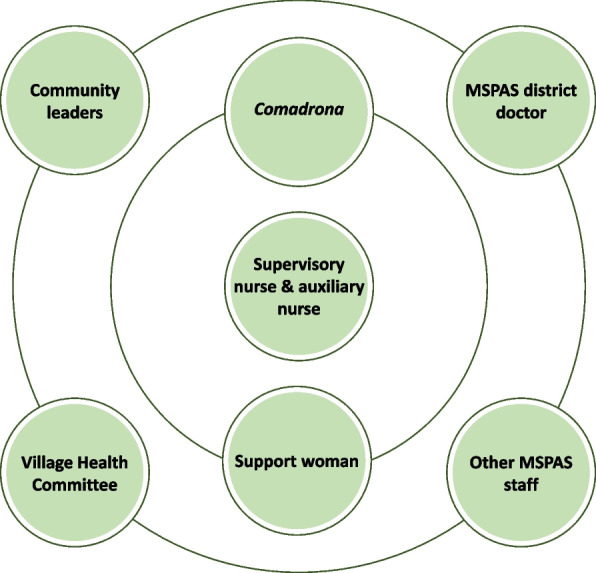
Circles of support that Birthing Center staff use to convince a family to accept a referral

#### Decision level 3: whether and how the staff should manage a complication if the family refuses the referral

If a family rejects a referral, the Birthing Center staff must then decide whether to attempt to resolve the complication on-site, knowing it is preferable to refer the patient. The threat to the life of the patient and her neonate are considered. Lower-level staff rely on support from higher-level staff and MSPAS doctors to reduce the risk to the mother and her neonate. The staff also consider the effect that the loss of life may have on the relationship between the Birthing Centers and the MSPAS. To mitigate potential backlash to the Project, the staff prepare release waivers and engage and incorporate the MSPAS and area officials in the decision-making process.

#### Decision level 4: what the family will do if the birthing center staff are unwilling to provide further care

Finally, if the family refuses the referral and the staff determine that they cannot resolve the complication on-site, the family must decide whether to accept the referral and go to the referral facility or to take the woman home without care. Records confirm staff accounts that families returning the mother home without care is a rare event, with one reported case occurring out of the 122 referrals made. Still, rejected referrals pose a threat to the future of the Birthing Centers and safety of mothers in the Project Area.

#### Other factors

Multiple factors encourage or discourage not only the acceptance of a referral throughout these four decision points but also the preparation of the woman and her family for the delivery. The Birthing Centers make up only part of the overall CBIO+ Approach of Curamericas/Guatemala aimed at reducing maternal and neonatal mortality. Community education and health outreach in partner communities provided through Care Groups and prenatal classes provide a strong foundation of influence, trust, and education that help women to learn about the importance of having a facility-based birth; to recognize and respond to danger signs during pregnancy, birth, and the postpartum period; to learn about the roles and limitations of the Birthing Center; to prepare a birth plan; and to learn about opportunities to reduce costs in case a complication arises by buying transportation insurance through the Birthing Center.

Women from non-partner communities who came to a Birthing Center were more likely to be referred to a hospital, and women from non-partner communities who were referred to a hospital were more likely to undergo a cesarean section than women from partner communities who came to a Birthing Center (data not shown). This suggests that women from non-partner communities were more likely to use a Birthing Center after a complication had started to develop and that these women and their families may misunderstand the function of the Birthing Centers, presenting only after a complication has occurred, leading to more serious complications and worse outcomes.

#### Emotional impact on staff

Even though the Birthing Centers have been successful in resolving many complications the staff would have preferred to refer, this decision is often accompanied with considerable fear for the well-being of both the mother and her neonate as well as with anxiety about the future of the Birthing Centers if the mother or neonate were to die in their care. Throughout the interviews it was apparent that staff undergo significant stress and anxiety when they manage complications in the Birthing Center. Even when a patient is referred, staff expressed that they wait in fear until they hear that the patient’s family followed through with the recommendation and that the complication was successfully resolved. Additional anxiety stems from fear of repercussions from the MSPAS. Multiple staff members mentioned that the Birthing Centers are under scrutiny by the MSPAS and so an error on their part, or even an unavoidable death on their hands, could have a significant, negative impact on the Birthing Centers﻿ and their ability to continue functioning. The staff’s concern about the well-being of both the mother and her neonate as well as about the future of the Birthing Centers acts as a deterrent to their trying to resolve cases outside of their capacities in the Birthing Center and increases the pressure involved in convincing the family to accept the referral.

### Implications of the findings

This article highlights the capacity of Birthing Centers staffed with lower-level health staff without formal midwifery training to manage obstetrical complications in isolated, rural Guatemala, where referral care is at least 4 h away and the barriers to referral are substantial. These Birthing Centers are often faced with difficult decisions about the management of complications when they occur. We show here that the Birthing Centers have been effective in managing these complications in a way that protects the life of the mother and neonate while operating within local constraints. The success that the Birthing Centers have achieved in the management of complications reflects the valuable training that the staff received from the highly experienced supervisory nurse – during both the initial training and frequent ongoing continuing education.

Indigenous women throughout the world often seek culturally relevant, respectful readily accessible and affordable care. They generally consider care from the formal health system as being only for complications and emergencies [34, 35]. Common causes elsewhere in Guatemala for not accepting recommendations for referral include (1) opposition from the husband or other family member and (2) difficulties (including costs) in arranging transport [36]. Experiences have been reported from the central highlands of Guatemala with obstetric care navigators, who are community-based workers employed to accompany Indigenous mothers in need of referral to a hospital to provide support and to improve the patient experience with facility-level obstetric care [35, 37]. Support Women at the Birthing Centers in our Project Area also have performed this role.

The types of peripartum complications encountered by the staff at the Birthing Centers are consistent with the global experience. The overall peripartum complication rate during the study period was 15.3%, which is consistent with the global experience – approximately 15% of women will experience a complication in the peripartum period [38]. Globally, the percentage of complications that are caused by post-partum hemorrhage, pre-eclampsia/eclampsia, retained placenta, and obstructed labor are broadly consistent with data from the Birthing Centers (Table 7).

**Table 7:** Comparison of the incidence of peripartum complications globally with that among women obtaining care at a Birthing Center

Type of peripartum complication	Percentage of complications at the Birthing Centers	Comparable percentage based on global data
Labor dystocia/obstructed labor	22%	9% [39]
Post-partum hemorrhage	17%	10% [40]
Pre-eclampsia/eclampsia	11%	4% [40]
Retained placenta	6%	3% [41]

Global estimates need to be viewed with caution, however, given the lack of standard definitions, and the vast heterogeneity of health systems that make maternal morbidity very difficult to accurately measure and compare across settings [42]. In addition, most studies of birth complications are facility-based and may not be applicable to a birthing center setting [42, 43]. Studies of birthing center complication rates are almost all from high-resource settings, and again may not be applicable to this rural, low-resource setting. When compared to the limited data available from similar settings, the Birthing Center complication rates are consistent with experiences elsewhere [44–47].

A key, highly unanticipated finding was the high percentage of complications (42.2%) that were resolved in the Birthing Centers*,* which are meant to be places for normal deliveries and for the timely stabilization and referral of complications. Although limited documentation is available, records show that out of the 211 complications, at least 38 families initially refused referral to a higher-level health facility. In some cases, the staff agreed to do their best to manage the complication at the Birthing Center*.* In many of these instances, mothers presented late in their labor course, with delivery imminent, leaving no time for referral.

An assessment of the complications resolved at the Birthing Centers versus those referred indicates a high level of clinical judgment and skill on the part of the Birthing Center staff. For example, the Birthing Centers do not have the capability to properly manage pre-eclampsia/eclampsia, and thus 95.8% of pre-eclampsia/eclampsia cases were referred to a higher-level facility for management. In contrast, the Birthing Center staff do have the necessary training and medications to manage retained placenta; they resolved 84.6% of cases of retained placenta. Thus, our data indicate that the staff are using sound judgement in their decision-making processes regarding referrals and are skilled at resolving complications.

### Other reported experiences with community birth centers in isolated settings of low-income countries

While there is extensive published literature on Birthing Centers for low-risk deliveries in high-income settings staffed by formally trained midwives, we have not been able to identify any studies similar to ours that assess the management obstetrical complications by lower-level health staff in an isolated, rural area of a low-income country. Although the existence of such facilities is not uncommon, more published, peer-reviewed studies about the services they provide and the management of complications is needed. We are aware of only one other study describing community ownership of a Birthing Center, but this was for a maternity waiting home in Zambia [48]. A review of birthing centers in informal urban settlements of low-income countries has also recently been published [49].

### Limitations

Chief among the shortcomings of the study is that we did not interview mothers and families directly regarding their perceptions of the Birthing Centers and their acceptance or rejection of referrals, instead relying on the Birthing Center staff as a secondary source. Quantitative data on complications were obtained from registry records drawn from the Birthing Centers’ clinical delivery records. Any complications not recorded in these records or that occurred after discharge of the mother would not have been included, so complications may have been underreported. We also were not able to retrospectively quantify the exact percentage of referrals that were ultimately rejected, since this was not always indicated in the Birthing Centers*’* clinical records. Finally, we have included in our analysis only maternal complications and their management. Further analysis of management of complications of newborns and the mortality of those newborns born at Birthing Centers compared to those born at home would have been useful, but such information was unavailable.

## Conclusion

Despite the challenges of accessing high-quality maternity care in the rural, isolated mountains of Guatemala, the Birthing Centers supported by Curamericas/Guatemala succeeded in reducing maternal mortality in a small catchment of 26 communities with a population of 8,702 people. Central to this success has been the management of obstetric complications by Birthing Center staff, wherein staff members have resolved a large percentage of complications on-site and have successfully referred almost all other cases to higher-level care. A single maternal death among all 1,378 laboring women cared for at a Birthing Center between 2009 and 2016 indicates an MMR of only 72.6. This is less than half the MMR for all Indigenous Guatemalan women (166), 75% lower than that estimated for Huehuetenango (277) [23], and only one-sixth of the risk we have documented for women living in the Project Area [5]. We conclude that the Birthing Center staff are able to provide skillful and thorough attention to complications.

Despite these successes, we found that consistent barriers to families accepting referrals persist. These include: (1) inaccessibility of higher-level health facilities due to cost and distance; (2) discrimination and lack of cultural sensitivity experienced at higher-level health facilities; and (3) cultural traditions and preferences including a lack of women’s decision-making autonomy, low valuation of women’s lives, and traditional preference for home delivery. All of these barriers must be addressed systemically to improve family compliance with referrals and to ultimately eliminate readily preventable maternal deaths in this population.

To conclude, results from this study provide insight into the Birthing Centers’ success toward preventing maternal mortality and may be used to improve future initiatives both in Guatemala and in rural areas around the world still struggling to reduce maternal mortality. The dynamics of managing maternal complications remain a challenge. The many barriers to referring obstetric complications need to continue to be addressed in order to ensure that these women obtain the high-quality care that is their fundamental human right.

## Data Availability

All of the Project reports, de-identified data, as well as publications about the Expanded CBIO+ Approach cited in this article are available from the corresponding author on request.

## Abbreviations

CBIO: Census-Based, Impact-Oriented Approach
Community Birthing Center: *Casa Materna Rural*
EMT: Emergency medical technician
FGD: Focus group discussion
MMR: Maternal mortality ratio
MSPAS: *Ministerio de Salud Pública y Asistencia Social* (Ministry of Public Health and Social Assistance)
NGO: Non-governmental organization
